# A comprehensive review of integrated management strategies for damping-off disease in chili

**DOI:** 10.3389/fmicb.2024.1479957

**Published:** 2024-10-17

**Authors:** Chen Delai, Ghulam Muhae-Ud-Din, Rimsha Abid, Tian Tian, Ruirui Liu, Yan Xiong, Shirong Ma, Abazar Ghorbani

**Affiliations:** ^1^School of Agriculture and Bioengineering, Longdong University, Qingyang, China; ^2^Gansu Key Laboratory of Protection and Utilization for Biological Resources and Ecological Restoration, Qingyang, China; ^3^Department of Plant Pathology, College of Agriculture, Guizhou University, Guiyang, China; ^4^Institute of Plant Protection, MNS University of Agriculture, Multan, Pakistan; ^5^National Key Laboratory of Green Pesticide, Key Laboratory of Green Pesticide and Agricultural Bioengineering, Ministry of Education, Center for R&D of Fine Chemicals of Guizhou University, Guiyang, China

**Keywords:** biocontrol, chili cultivation, epidemiology, *Fusarium* spp., *Pythium* spp., resistant cultivars, sustainability

## Abstract

Damping-off disease in chili (*Capsicum annum* L.) cultivation is a significant global issue, severely affecting seeds, seedlings, and young plants, regardless of the location of cultivation, whether in greenhouses or open fields. Despite chili being a widely popular vegetable used in various cuisines globally, farmers face challenges in meeting the growing demand due to the extensive damage caused by this disease, ranging from 20 to 85%. The shelf life and quality of mature pods are also severely affected. Damping-off disease is mainly caused by soil-borne fungus from the *Pythium* species, with additional contributions from *Phytophthora*, *Fusarium*, and *Rhizoctonia* species. These pathogens’ adaptability to diverse environmental conditions and resistance to synthetic fungicides make controlling damping-off on a commercial scale challenging. However, integrated disease management has shown promising results as a remedial approach. In this review, we discuss the current state of chili diseases, the nature of the pathogens causing damping-off, the epidemiology of the disease, and various control mechanisms. In this review, we broadly discuss the current state of chili diseases, the nature of the pathogens causing damping-off, the epidemiology of the disease, and various control mechanisms. Furthermore, we highlight the importance and efficacy of integrated disease management techniques, along with future prospects in unexplored areas, such as host–pathogen interaction and sustainable disease control measures. The information in this review aims to assist chili growers in understanding the epidemiology and management of damping-off in chili cultivation.

## 1 Introduction

Chili (*Capsicum annum* L.) is a member of the Solanaceae family and is considered a vital vegetable *cum* spice, which is one of the most extensively cultivated crops worldwide. Morphologically, this is a heavily branched herbaceous annual shrub that grows well in sandy, fertile soil. The flowers are terminal, pentamerous, solitary, and bisexual, with a structure that allows the corolla to rotate. The stamens have blue to purple anthers attached at the base of the corolla (Awang et al., 2013). Based on the fruit’s shape, color, pungency, size, flavor, and use, chili is classified as a perennial crop and can be grown throughout the year. Approximately 400 different cultivars of chilies are grown, and well-developed pods are used as food ingredients for spices, sauces, pickles (Zou and Zou, 2021), or beverages and medicine. In urban lifestyles, chili is grown as an ornamental plant in gardens. Chili contains a significant amount of minerals: potassium (K), iron (Fe), magnesium (Mg), and vitamins A and C, along with other significant nutrients (Sarkar et al., 2022).

Chili originated in the tropical and subtropical regions of Central and South America and was introduced to Zhejiang Province (China) in 1591. It is now distributed across northern, western, and southern China (Zou et al., 2020). China, Mexico, Indonesia, Turkey, and Spain are the leading green chili-producing countries, collectively responsible for approximately 75% of global production in 2013, according to FAOSTAT (2020). China, in particular, made the largest contribution, accounting for over half of the world’s chili production, with a cultivation area exceeding 21,474 km^2^ in 2019 (Zou and Zou, 2021). This surpasses the current land area allocated to versatile vegetables in the Chinese market. Moreover, more than 3,070 km^2^ is cultivated in Guizhou province (26.5982° N, 106.7072° E), which is higher than in any province in China.

Different crop genotypes are found in China, with productivity varying across provinces. The total chili yield in 2019 exceeded 64 million tons, which accounted for 7.76% of China’s total national vegetable output. The total pepper yield in China reached 250 billion CNY, accounting for 11.36% of the total output value of national vegetables and contributing to 1.14% of the total farm income (Zou and Zou, 2021). Though there is a rising demand for chili for versatile purposes, today, the supply chain has failed to cater to existing demand due to various challenges in chili cultivation. Among them, biotic stresses such as pest and disease attacks and abiotic stresses such as temperature fluctuations, water scarcity, and salinity are crucial. In addition, pre- and post-harvest losses due to poor management practices occurred on various scales.

Chili plant death in nurseries due to damping-off caused by pathogens has become a major issue in chili cultivation worldwide. The main causal organism is a soil-borne pathogenic fungus, *Pythium* spp., which affects chili plants in seedlings and younger roots. In addition, *Rhizoctonia solani*, *Fusarium,* and *Phytophthora* spp. are considered other pathogenic organisms that cause damping-off (Rini and Sulochana, 2006). In this study, we have thoroughly discussed the damping-off disease in chili cultivation, current yield losses, sustainable crop management practices, integrated pest control methods, advancements in disease control technology, and its prospects.

## 2 History and distribution of the host crop, *Capsicum annuum* L.

*Capsicum annuum* L. has been known for over 9,500 years. Chili is a native crop of Southern America and was first cultivated in Peru at approximately 7,500 BC (MacNeish, 1964). First, three species of chili, i.e., *C. chinense*, *C. frutescens*, and *C. annuum,* developed from common ancestors that grew widely in the north of the Amazon basin (NW-Brazil, Columbia), spreading to the other parts of America. Then, two more species, i.e., *C. baccatum* and *C. pubescens,* developed in different parts of America at the end of the eighteenth century (IBPGR, 1983). The introduction of chili to China is endorsed by the journey of Columbus, who took the chili seeds from Spain, introduced them to Europe, and spread them to the subcontinents of Asia and Africa (Heiser, 1976). Columbus confused the pungent fruits of *Capsicum* with black pepper *Piper nigrum* L., calling them red pepper owing to the red-colored fruits. *Capsicum* is not only related to the *Piper* genus. However, the terminology surrounding *Capsicum* is often confusing. Names such as chili, chili, aji, chile, pepper, and paprika denote the pungent fruits of *Capsicum*. The crop status spread quickly across Europe, moving to China, Japan, India, and Pakistan. Unlike other important cuisines, it became one of the most important cuisines in Asia and Europe (Zou and Zou, 2021).

While there are reports of 20 wild species of chili peppers, only five species are currently used for cultivation: *C. annuum, C. baccatum, C. frutescens, C. chinense, and C. pubescens.* Among these, *Capsicum annuum* var. *annum* is the most extensively cultivated and economically significant among domesticated chili peppers. Notably, it is the only species commercially grown outside America, in Asia and Africa. Particularly in China, the crop was introduced to Zhejiang Province in 1591, and then it became the most important ingredient in various cuisines throughout the country (Zou and Zou, 2021). The common name for chili in China is “Lajiao” in Chinese. The bell pepper is the non-pungent fruit of *Capsicum* and is known as “Tianjin or Caijiao” in Chinese. Over 95% of crops cultivated in China are *C. frutescens* L., *C. annuum* L., and *C. chinense* Jacq (Zou and Zou, 2021), which are mostly cultivated in the northern and southern parts of China.

## 3 Global production and Chinese contribution

Chili peppers are widely grown and consumed worldwide, with larger shares in Asia, Africa, America, and Europe. China is one of the major consumers, producers, and exporters of chili peppers (Sundari et al., 2023). China’s share of global chili pepper exports was approximately 13%. In 2020, global production was estimated at 37.9 million tons, with China accounting for approximately 36%. Mexico and Indonesia were the second and third largest green chili producers. According to the Guinness World Records and the Food and Agriculture Organization (FAO), China produces approximately 46% of the global chili supply to the market, as mentioned in Table 1 (FAOSTAT, 2020; Sachin et al., 2022).

**Table 1:** Annual chili production by the world’s top producers in million tons (FAOSTAT, 2020).

Country	Annual chili production in thousands of tons
Green chili	
China	16,836
Mexico	2,818
Indonesia	2,773
Turkey	2,637
Spain	1,473
Egypt	867
Nigeria	767
Algeria	718
USA	566
Netherlands	430
Dry chili	
India	1702
Thailand	322
China	308
Ethiopia	296
Bangladesh	158
Pakistan	141
Myanmar	140

The growth in chili exports worldwide has been driven by production surpluses and the significant potential of the global chili trade. Since 2004, the volume of chili exports has experienced consistent growth, with an average annual growth rate of 6.04% (Shimbov et al., 2019; Sundari et al., 2023). However, interpreting global chili production trends and China’s role in the industry is essential for policymakers, researchers, and stakeholders involved in the chili trade (Yin et al., 2022).

## 4 Uses and importance of chili

Chili is utilized in various forms, including fresh, green, or ripened fruits, along with dried and powdered forms. Fresh green pungent chili fruits are generally used in stuffing, salads, pickles, and as a flavoring agent in cooked meals, while fresh green non-pungent fruits are used as vegetables or processed with other food items for flavor. Additionally, highly pungent fruits are used as a spice to stimulate appetite and as a flavoring agent in ketchup. In addition to culinary uses, small quantities of chili are used in the cosmetic and garment industries (Saxena et al., 2016).

Chilies have the potential to alleviate micronutrient deficiencies by supplying vitamins A, C, E, and B, along with minerals such as molybdenum, manganese, folate, potassium, and copper. They also supply significant macronutrients, such as protein, carbohydrates, fats, and dietary fiber, which are essential for human health (Olatunji and Afolayan, 2018).

Peppers contain a wide range of phytochemicals, such as phenolics and flavonoids, which possess important antioxidant activities that help reduce the risk of degenerative diseases (Saleh et al., 2018). Additionally, capsaicin in chili improves digestive health and nutrient absorption (Marini et al., 2015; Rosca et al., 2020), provides relief from joint pain (Fattori et al., 2016), exhibits anti-inflammatory properties (Chung and Campbell, 2016; Fattori et al., 2016), and inhibits the growth of several types of fungi, including *Candida albicans* (Behbehani et al., 2023).

Hot chili peppers also have antiviral properties, which can help prevent colds and flu while boosting the immune system (Maurya and Sharma, 2023). Regular consumption of chili fruit is helpful against anorexia, varicose veins, hemorrhoids, and liver congestion. Chili extracts, both in pure and processed forms, are used externally as analgesic rubefacients for treating rheumatism, back pain, muscle pain, and articular and swollen feet, and are even used as antidotes for poisoning. Additionally, chilies have non-food and non-pharmacological uses, such as in the preparation of “pepper sprays” for self-defense and in making “natural and organic pesticides.”

## 5 Chili plant morphology

The chili plant displays unique morphological features. The chili plant is a vigorously branched herbaceous species with primary, secondary, and tertiary branching. Its height typically ranges from 50 to 100 cm (Pawaskar, 2023). Its solitary, often paired, bisexual flowers have bell-shaped, twisted corollas with 5–6 lobes. Notably, chili plants are primarily cross-pollinated by insects, with natural rates reaching up to 50% (Gao et al., 2023). Chili peppers are distinctive berries with seeds not embedded in the pericarp (Azlan et al., 2022). They change from green to red or purple upon ripening, with variations in shape, size, and pungency. Pungency is determined by capsaicin content and genetics, while the red color comes from capsanthin (Wang et al., 2023). Orbicular seeds are developed within the fruit, and their size is influenced by nutrition (Barboza et al., 2022). Chili plants, which are highly branched and have shallow roots, exhibit drought tolerance but struggle in waterlogged soil (Supplementary Figure S1; Guo et al., 2022).

### 5.1 Favorable climate for chili and chili diseases

Chilies thrive in tropical and subtropical regions up to 2000 m in altitude, excluding pungent varieties (Aashish et al., 2022). Optimal conditions include warm, humid climates that promote growth and fruit maturity, with an ideal annual rainfall of 850–1,200 mm. However, excessive rain and humidity can lead to poor fruit set and fruit rot. The ideal temperature for chilies ranges between 20°C and 25°C during the day and 15°C and 21°C at night (Arom, 2022). A day length of 9–10 h stimulates plant growth, boosting productivity by 21–24% and enhancing capsicum quality (Kramchote and Suwor, 2022).

Soil is the main substrate for chili in open field conditions, and various other solid substrates, such as grow bags, are used under greenhouse conditions. Chili prefers well-drained, aerated soils rich in organic matter. Ideal soils are light loamy or sandy loamy with lime and organic content, while light soils require irrigation and organic fertilization. The 6–7 pH range is ideal for optimum growth, and higher salinity hampers plant growth. Chili diseases have been a major reason for yield reductions in the world. Diseases caused by bacteria, viruses, fungi, and nematodes have badly affected chili crops worldwide (Su et al., 2023; Xie et al., 2023). Diseases associated with chili crops are summarized in Table 2. One such fatal disease is damping-off, which occurs by soil-borne fungi genera, whether occurring pre- or post-plant emergence, accounting for up to 90% plant death and 62% seedling viability loss in nurseries and fields, as originally documented by Majeed et al. (2018).

**Table 2:** Biotic and abiotic stress of chili reported from different parts of the world.

Disease name	Pathogen/abiotic stress	Symptom	Crop stage	Plant parts affected	Countries affected	Reference
Fungal diseases						
Damping off	*Pythium* spp. *Fusarium* spp. *Sclerotinia* spp.	Failure of seedlings to emerge, soft, water-soaked, and discolored area at the base of the stem, stem constriction, seedling death, brown and rotted roots	seedling after transplantation	Roots and crowns of older plants	Worldwide	Hyder et al. (2021)
Black mold	*Alternaria alternata*	Rotting of fruits	Fruiting	Fruits	Tropical and sub-tropical countries	El-Garhy et al. (2020)
Phytophthora root rot	*Phytophthora capsici*	Rapid yellowing, wilting, soft rot, collapse of the rot, yellow-green needles, wilting, slow growth, dead branches, tree death	Seedling, fruiting	Roots	New Mexico	Sanogo and Carpenter (2006)
Verticillium wilt	*Verticillium dahliae*	Wilt, chlorosis, anthocyanescence, stunted and/or distorted growth, necrosis, and premature plant senescence.	Seedling	Leaves, stem, roots	New Mexico	Sanogo (2003)
Rhizoctonia root rot	*Rhizoctonia solaniis*	Stunting, yellowing of lower foliage, stem discoloration, reddish-brown dry cortical root rot, snapping off during high winds, reduced nodulation, yellowing or wilting of leaves	Seedling	Stem, roots	New Mexico	Sanogo (2003)
Frog eye leaf spot	*Cercospora capsici*	Circular lesions with a white canter, frog-eye, blighting of foliage, water-soaking of leaves, yellowing, reduced fruit size	Mature stage	Leaf	Taiwan	Salam et al. (2022)
Powdery mildew	*Leveillula taurica*, *Oidiopsis taurica*	White, powdery, yellow, or chlorotic or powdery growth appearance on the upper leaf surface	Flowering and fruiting	Leaf	Places with a warm and dry climate	Glawe et al. (2010)
Anthracnose/ripe rot	*Colletotrichum* spp.	Leaf spots, fruit rot, stem cankers	Fruiting	Fruit	Tropical and sub-tropical countries	Than et al. (2008) and Saxena et al. (2014)
Fruit rot	*Colletotrichum truncatum/capsici*	Sunken spots, collapse, discoloration	Mature plants	Fruit	Tropical and sub-tropical countries	Saxena et al. (2016)
Fusarium wilt	*Fusarium solani* var*. capsici*	Yellowing and wilting, brown discoloration of the stem, and rotted roots	Seedling after Transplantation	Leaf	Leaf chlorosis, vascular discoloration, and wilting	Suryanto et al. (2010)
Bacterial diseases						
Bacterial leaf spot	*Xanthomonas campestris* pv. *vesicatoria*	Water-soaked spots, dark brown lesions, yellowing and dropping of leaves, deformation and twisting of young leaves	Seedling, flowering, fruiting	Leaf, stem, and fruits	Tropical and sub-tropical countries	Vauterin et al. (2000) and Abbasi et al. (2002)
Bacterial soft rot	*Erwinia carotovora* pv*. carotovora*	Watery lesions, tissue softening, sunken, water-soaked lesions, and wilting	Fruiting	Fruit	Areas with wet and cold climate conditions	Stommel et al. (1996)
Bacterial wilt	*Ralstonia solanacearum*	Yellowing, wilting, stunting, vascular necrosis, and vascular browning	After transplantation	Root and stem	Tropical and sub-tropical countries with high rainfall	Nguyen and Ranamukhaarachchi (2010)
Bacterial canker	*Corynebacterium michiganense*	Water-soaked margins and circular tan to dark spots on leaves, brown cankers on stems, and leaf curling,	Leaf and fruit	Leaves and stem	United States of America	Lewis Ivey and Miller (2000)
Viral diseases						
Beet curly top virus	*Leafhopper transmitted Geminivirus*	Puckering, curling, yellowing, and stunting	Maturity	Whole plant	Western United States, Eastern Mediterranean basin	Bahari et al. (2022)
Pepper mottle virus	*Aphid transmitted Potyvirus*	Mild or bright yellow mottling on the leaves, leaf distortion, yellowing and stunting, blistering, and necrosis on fruit.	Maturity	Leaves and fruits	Florida, Southern USA, Arizona, Central America, Mexico, Thailand and India	Kaur et al. (2014)
Alfalfa mosaic virus	*Aphid transmitted* the *Bacilliform virus*	Mottled leaves, irregular light and dark green areas on leaves	Maturity	Leaves	New Zealand	Fletcher (1983)
Pepper leaf curl virus	*Whitefly transmitted Geminivirus*	Upward curling of leaves, yellowing of veins, leaf size reduction, stunted growth, and yield reduction.	Maturity	Leaves and stem	United States, India, Nigeria, and South Asian countries	Chattopadhyay et al. (2008)
Pepper veinal mottle virus	*Aphid transmitted Potyvirus*	Stunting, curling, or twisting and yellow mosaic on the leaves, vein clearing, curling, twisting, and puckering of leaves and fruits.	Maturity	Leaves and fruits	Asian countries	Moury et al. (2005)
Pepper mosaic virus	*Aphid transmitted Potyvirus*	Mosaic and curling on leaves, fruit distortion, mottling symptoms on the leaf stem and fruits	Maturity	Leaf, stem, fruits	Argentina	Ahn et al. (2006)
Chili veinal mottle virus	*Aphid transmitted Potyvirus*	Dark green banding along veins on leaves, distorted leaves with mosaic patterns, twisted or fallen leaves, vein banding, reduced fruit size, severe puckering, thinning, shoestring, and cupping of leaf lamina in king chili plants, stunted growth with dark-green streaks on stems, mottled and deformed fruit, most flowers drop before fruit sets	Maturity	Leaf and fruits	Asian countries	Moury et al. (2005)
Nematode diseases						
Root-knot nematode	*Meloidogyne incognita*	The prominent symptoms are of silting, yellowing, gall formation in roots, and stunted plants.	Maturity	Roots	Different parts of the world	Thiyagarajan (2014)
Stubby root nematode	*Paratrichadorus minor*	Stunted, chlorotic, wilted, and symptoms resemble nutrient deficiency.	At any stage	Roots	North America	Li et al. (2010)
Sting nematode	*Belonolaimus longicaudatus*	Stunted, chlorotic, wilted, and symptoms resemble nutrient deficiency	At any stage	Roots	North America	Shaver et al. (2017)
Root-lesion nematode	*Pratylenchus penetrans*	Stunted, chlorotic, wilted, and symptoms resemble nutrient deficiency, low flowering, and yield.	At any stage	Roots	North America	Figueiredo et al. (2021)
Insects and mite infection						
Mite feeding injury	*Polyphagotarsonemus latus*	“Inverted spoon-shaped” leaves, pods with a rusty/corky surface	Seedlings and maturity	Leaves and fruit	Africa, Asia, Australia, South and North America, Pacific lands	Venzon et al. (2008)
Aphid feeding injury	*Myzus persicae Aphis gossypii*	Distorted, mottled young leaves, chlorosis, leaf drop, reduced fruit size	Seedlings and maturity	Leaves and fruit	India, Orient and Pacific lands, Sri Lanka, and Continental USA	Tapia et al. (2008) and Varghese and Mathew (2013)
Thrips feeding injury	*Thrips parvispinus Scirtothrips dorsalis*	“Boat-shaped” curled leaves, distorted pods	Seedlings and maturity	Leaves and fruit	Worldwide	Maharijaya et al. (2011) and Johari et al. (2014)
Abiotic stress						
Blossom-end rot	Watering and calcium deficiency	Light green or yellow-colored sunken spot and water-soaked area that becomes a dark brown or black dry rot	At any stage	Fruit	Different parts of the world	Marcelis and Ho (1999)
Sunburn	Sun	White or black discoloration on fruits, brown or white foliage, leaves turning brown or ivory-white and becoming dry and crispy, yellow, bronze, or brown spots on the sun-exposed side of the fruit	At any stage	Fruit	Tropical and sub-tropical countries	López-Marín et al. (2011)
Salt injury	Salt	Stunting or death of seedlings, reduction in leaf, flower, and fruit size, necrosis of leaf margins, less vegetative growth, reduced root growth	At any stage	Seedling	All over the world	Mustafa et al. (2014) and Butt et al. (2021)
Wind injury	Wind	Wilting and death of young seedlings, broken stems, or branches	At any stage	Foliage	All over the world	Pohronezny et al. (1992)
Hail injury	Hail	Severe hail injury results in shredded leaves or defoliation-damaged stems and fruit and physical damage to the foliage	At any stage	Foliage and Fruit	All over the world	Joukhadar and Walker (2020)
Herbicide injury	Herbicide	Leaf cupping or curling, petiole twisting, epinasty, blotches or discoloration on the leaves, change of leaf color, severe tissue distortion or deformation	Maturity	All parts of the plant	All over the world	Galloway et al. (2000)
Nutrient deficiencies and toxicities	Nutrients	Stunted growth, chlorosis, interveinal chlorosis, and purplish-red discoloration or premature dying of leaves	At any stage	All parts of plants	All over the world	Khaitov et al. (2019)

## 6 Damping-off disease on chili

Damping-off is a significant fungal disease affecting chili production worldwide, leading to substantial economic losses. The disease primarily targets seedlings, causing poor germination and plant death, which reduces overall crop yield. Farmers experience financial setbacks due to the need for replanting, increased use of fungicides, and lower productivity. In regions highly dependent on chili cultivation, such as China, India, and Mexico, the disease poses a significant threat to growers’ livelihoods, disrupting both local and global markets. The chili crop is infected by more than 100 different types of plant pathogens during its vegetative and reproductive stages (Jayapala et al., 2019). Damping-off disease is caused by soil-borne pathogens such as *Pythium* sp., *Phytophthora* sp.*, Rhizoctonia solani, and Fusarium* spp. (Thakur and Singh, 2023).

Damping-off is one of the most critical diseases that leads to the decay of germinated seeds and young seedlings, which causes a huge economic loss for farmers in nurseries and fields. These fungal or fungal-like organisms cause seed death during germinating in nurseries, with losses of up to 90% under pathogen-favorable conditions (Arora et al., 2021). Damping-off on chili occurs in two stages: the pre-emergence and post-emergence phases. In the pre-emergence phase, disease typically begins during the seedling stage, where the fungi invade seeds or young plants in waterlogged or poorly drained soil. Initial symptoms include seed rot or germination failure, leading to poor crop emergence. Infected seedlings may develop water-soaked lesions at the base of the stem, which gradually turn brown, causing the stem tissue to collapse. This pre-emergence damping-off stage is particularly destructive, as the seedlings die before breaking the soil surface (Majeed et al., 2018). However, the stem tissue near the soil line becomes soft, sunken, and girdled during the post-emergence phase. The affected seedlings wilt, fall over, and die due to disrupted nutrient and water transport, a condition often referred to as “wire stem” (Lamichhane et al., 2017).

This deterioration often results in reduced seedling vigor, and the seedlings may die near the coleoptile, exposing whitish fungal growth on the surface. In the initial stages, affected plants exhibit wilting, eventually leading to a severe infestation. Surviving plants display stunted growth, and the affected areas often exhibit irregular and uneven development (Were et al., 2023). Damping-off symptoms can generally be observed from seeding until the 4th to 6th week post-sowing (Supplementary Figure S2). In this study, we focus mainly on *Pythium* spp. (Lamichhane et al., 2017) regarding damping-off. Several species of *Pythium* associated with chili to cause damping-off disease in different parts of the world are mentioned in Table 3.

**Table 3:** Fungal species associated with damping-off of chili in different parts of the world.

Pathogen	Country/Province	Reference
*Pythium cedri*	Jiangsu/China	Rai et al. (2020)
*Fusarium oxysporum*	Guizhou/China	Luo and Yu (2020)
*P. helicoids*	Guangdong/China	Chen et al. (2016)
*Pythium* spp. *Fusarium* spp., *Phytophthora* spp.	Heilongjiang/China	Dai et al. (2013)
*Pythium* spp.	Hainan/China	Hyder et al. (2021)
*P. breve* and *P*. *baisense*	South China	Long et al. (2012)
*P. myriotylum*	Pakistan	Hyder et al. (2021)
*P. aphanidermatum*	India	Muthukumar et al. (2010)
*P. graminicola*	India	Dubey et al. (2019)
*P. ultimum*	India	Zagade et al. (2016)
*P. spinosum*	Pakistan	Nawaz et al. (2016)
*P. diliense*	India	Muthukumar et al. (2018)
*P. heterothallicum*	India	Muthukumar et al. (2018)
*P. debaryanum*	Pakistan	Shahid et al. (2017)
*P. intermedium*	Pakistan	Nawaz et al. (2017)
*P. debaryanum*	India	Gomathi et al. (2012)

### 6.1 The biology of different pathogens causing damping-off

*Pythium* species are a group of soil-borne pathogens known for causing damping-off and root rot in a wide range of plants, including chili. They thrive in wet, poorly drained soils and attack seedlings, leading to stunted growth and plant death. Some *Phytophthora* species can cause damping-off in seedlings. For instance, *Phytophthora capsici* is known to cause damping-off in seedlings of various crops, including peppers and other vegetables. The pathogen attacks young seedlings, leading to poor germination, stem collapse, and death, similar to other damping-off pathogens such as *Pythium* and *Rhizoctonia*. *Rhizoctonia solani* is a soil-borne fungal pathogen that affects chili seedlings by attacking their roots and stems, leading to poor germination, wilting, and eventual death of the young plants. *Rhizoctonia* thrives in warm, moist conditions and can lead to significant crop losses if not managed effectively. *Fusarium* species are soil-borne fungi that attack chili seedlings’ roots and lower stems, leading to poor emergence, wilting, and seedling death. *Fusarium* thrives in warm, moist soils and can cause significant damage if conditions are favorable.

### 6.2 Epidemiology of the disease

Environmental factors play a key role in spreading the disease (Ghorbani et al., 2024a). The susceptible host, the chili plant, and the virulent pathogen, *Pythium* spp., along with conducive environmental conditions, are key elements for disease establishment. Temperature, moisture, and organic matter (Hansen and Keinath, 2013) have been directly linked with the incidence and severity of the damping-off owing to better establishment of the *Pythium* spp. with respect to attachment, establishment, and penetration into host tissues. Other environmental factors such as soil type, clay soil with high moisture holding capacity, rainfall intensity and duration, irrigation, humidity, and seedling surface witness are key factors responsible for damping-off disease. The pre-emergence damping-off requires 12°C while post-emergence damping-off require18-30°C and the optimum range lies from 24 to 30°C for successful infection of *Pythium* spp. (Dubey et al., 2019). Furthermore, a high soil moisture (Martin and Loper, 1999; Majeed et al., 2019) supports the dissemination of propagules and increases the size of the spermosphere, while a pH of ˃5.8 supports successful infection. The relationship between environmental factors with inoculum and crop geometry spread also leads to the possible development of disease (Arora et al., 2021). The detection and diagnosis of *Pythium* spp. pathogenic to chili plants involves utilizing both morphological characteristics and the analysis of specific DNA marker regions from the isolated pathogen. Recent research on *Pythium* spp. has predominantly focused on advanced molecular techniques based on PCR, including real-time PCR, multiplex PCR, loop-mediated isothermal amplification (LAMP) (Sridapan and Krajaejun, 2023), and internal transcribed spacer (ITS) analysis (Gaikwad et al., 2023).

### 6.3 Infection stages and disease cycle

*Pythium* spp. are eukaryotic organisms composed of filamentous, non-septate hyphae. Cell walls are primarily composed of cellulose and glucan, with limited amounts of chitin (Kiselev, 2020). *Pythium* spp. in soil is attracted to the exudates released from germinating seeds and developing seedlings, leading to seed rot and seedling death (Wohor et al., 2022). There are two main stages of *Pythium* spp. When the environmental conditions are favorable in the first stage, *pyrium* produces sporangia that grow into hyphae and vesicles with motile zoospores asexually. They are attracted to plant roots or other organic matter in the soil. In the other stage, *Pythium* reproduces sexually by combining an antheridium and an oogonium to form oospores, mostly during unfavorable environments. Sporangia can be single-celled or multi-celled and can be motile or non-motile.

**Figure 1:**
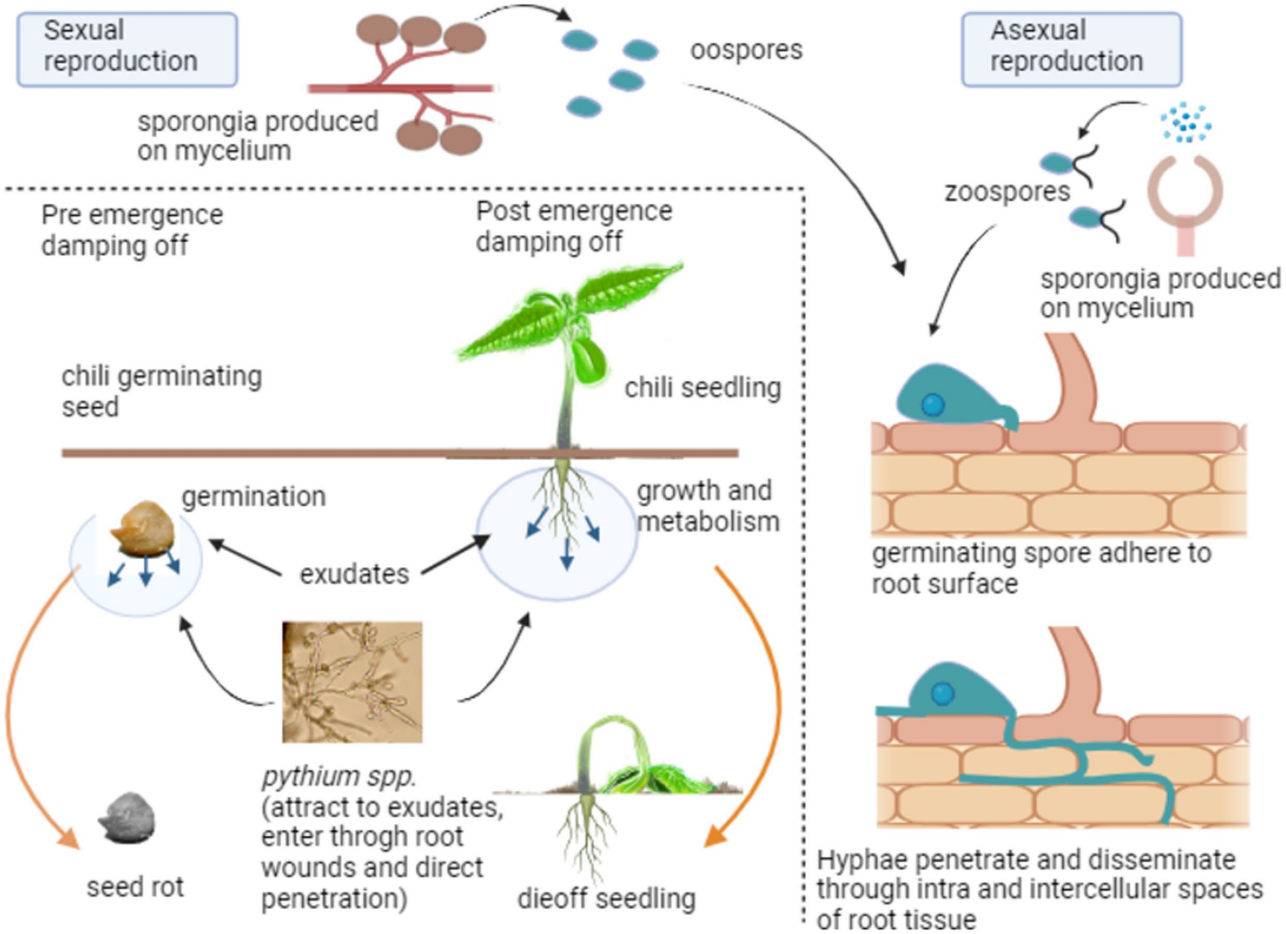
Infection stages and disease cycle of damping off disease.

Furthermore, *Pythium* spp. can be heterothallic or homothallic depending on the type of reproduction (Figure 1). When spores come into contact with a susceptible host, they can adsorb onto the root surface, forming a germ tube. The germ tube then penetrates the host’s cells via wounds, root tips, or direct penetration, grows to branch mycelium through intracellular and intercellular spaces, and absorbs nutrients while exhibiting pathogenic symptoms externally (Kushwaha, 2020). Sexual spores can thrive dormant in the soil in harsh environmental conditions as saprophytes and become active again when conditions become favorable (Parveen et al., 2020).

## 7 Integrated management of damping-off

Chemical control methods, such as applying pesticides and other synthetic compounds, have been widely used for managing various pests and diseases. While initially effective, these methods often face significant challenges, including developing resistance in target species, environmental contamination, and negative impacts on non-target organisms, including beneficial species (Ranjbar et al., 2023; Ghorbani et al., 2024b). Over time, repeated use of chemical agents can lead to diminishing returns, requiring higher doses for the same effect and exacerbating ecological damage (Ghorbani et al., 2023; Nanehkaran et al., 2024). Additionally, concerns regarding human health risks and ecosystem disruption further highlight chemical control’s limitations. These factors underscore the necessity of an integrated approach, combining biological, physical, and chemical methods to achieve more sustainable and resilient pest management strategies. Seed treatments are conducted to improve germination and seedling vigor (Bhattarai et al., 2022). There are physical, chemical, and biological seed treatment methods in use aiming at seed disinfection, disinfestation, and seed protection. Chemical seed treatment includes soaking seeds in thiram, captan, and brassicol at the rate of 2.5 g/kg (Misra et al., 2022), while physical methods include hot air, hot water, and electron treatments, compressed moist heat (Saha and Kumari, 2023). Biological seed treatment methods are neem leaf extract, garlic clove extract, ginger extract, and allamonda extract for chili (Mishra et al., 2020). The promising microbial seed treatment is carried out by treating chili seeds with *T. viride* and *P. fluorescence* (Mondal et al., 2022). Using synthetic seed treatments leads to phytotoxicity and germination defects, ultimately affecting human and animal safety. This is a disease preventive measure that can be taken before infestation from soil (Volynchikova and Kim, 2022).

Good agricultural practices follow guidelines such as sterilizing nursery soil using a combination of physical and biological methods, including solarization and the application of *Trichoderma* as a soil drench (Kumhar et al., 2022). Additionally, measures like burning soil with rice husks and bran, selecting soil from areas with no prior *Pythium* infestation history, using pure water and clean implements, using clean polythene sheets and organic amendments as mulch, fertilizing with organic materials from trustworthy, uncontaminated sources, and preventing water runoff from external sources are vital to avoid soil pathogen contamination (Zamoum et al., 2022). If symptoms of damping-off arise, the immediate uprooting and isolation of affected seedlings from the rest of the plants is necessary to prevent further spread. These practices collectively contribute to the successful adoption of best cropping practices.

In situations where the aforementioned preventive measures prove insufficient due to the severity of the infestation, the use of chemical control becomes a viable option. It is crucial to first assess the extremity of the disease within the nursery to determine the appropriate dosage of fungicide required. Before moving to chemical intervention, it is imperative to consider the soil’s overall health and the purity of the seeds selected for cultivation. More importantly, an immediate foundation of research outcomes to elucidate the morphology and physiology of *Pythium* and its response to external factors is needed to draw decisions. Fungicides should only be employed when all other methods have been exhausted, and the disease cannot be effectively managed or eradicated through alternative measures.

### 7.1 Use of chemical fungicides

Chemical control via fungicides is often considered a quick and effective way to prevent pathogens from infecting plant systems, particularly when applied to young, growing tissues like leaves, fruits, and flowers. This method is widely adopted due to the immediate results it yields compared to the lengthy process of developing resistant cultivars (Sawant et al., 2022). Farmers have resorted to various chemical alternatives to methyl bromide for soil fumigation, as non-chemical methods can sometimes be labor-intensive and less effective against soilborne diseases (Duniway, 2002). However, the use of chemical fungicides is constrained by a limited number of products registered for use on various crops and the high costs associated with these products, limiting chemical management of damping-off to a few active ingredients (Garzón et al., 2011). Fungicides from groups such as captan, benzimidazole, triazole, and dicarboximide have been recognized for their efficacy in controlling damping-off diseases (Lamichhane et al., 2016). Specifically, metalaxyl, tridiazole, and captan are effective against *Pythium* and *Phytophthora* species, while maneb and mancozeb work against *Pythium* and *Fusarium* species, and these are commonly used by growers (Lamichhane et al., 2016). Using systemic fungicides like metalaxyl before sowing can reduce *Phytophthora* and *Pythium* populations in the soil (Russell et al., 1990). For seed treatments, Satija and Hooda found that benlate (0.1%) and dithane M-45 were the most effective against *Pythium aphanidermatum* and *Fusarium solani* (Ansari et al., 1990). Soil drench applications of captan (Khrieba, 2020), as well as soil treatments with methyl bromide, solarization, and metalaxyl soil drench, have been successful in preventing *P. aphanidermatum* infection (Hickman and Michailides, 1998).

### 7.2 Recommended cultural practices

Cultural practices are vital in ensuring disease-free chili production with optimum yield. In detail, proper site selection, avoiding areas with a history of damping-off infection, ensuring good drainage, applying organic amendments to enhance soil quality, adopting the best cropping systems, and using healthy seeds are best cultural practices to avoid damping-off disease (Magnee et al., 2022). Further measures include infected plant parts being removed as soon as possible from the fields (Manjunatha et al., 2022), timely and adequate fertilizing, irrigation, mulching to regulate soil temperature and suppress weeds, implementation strategies to protect crops from pests and diseases, sterilization through solarization or seed treatment (Arora et al., 2022), cover cropping, green manuring, crop rotation, tillage, and managing proper space between crops (Moura et al., 2022). Soil physiochemical properties management, such as pH, CEC, and moisture, is also important. Combining these practices is the most effective approach to managing damping-off disease in chili crops (Cook and Haglund, 1991; Cook, 2001). Furthermore, enhancing seed vigor is crucial for controlling damping-off, even in high pathogen density (Lamichhane et al., 2017).

### 7.3 Use of botanicals and biological control agents

The utilization of botanical extracts presents a highly promising approach for managing chili damping-off disease caused by *Pythium* spp. Plant extracts, including neem, garlic, ginger, turmeric, lemon, and pepper, have been shown to possess inherent antifungal properties. Consequently, these botanical extracts can be a viable and eco-friendly substitute (Postma et al., 2003) for conventional chemical fungicides (Islam and Faruq, 2012; Hyder et al., 2021; Arora et al., 2022). However, it is essential to recognize that the effectiveness of botanicals can show variability dependent upon the particular plant species, the specific plant part used, and the method that was used for extraction (Arora et al., 2022). Moreover, the utilization of plant growth-promoting rhizobacteria (PGPR) in disease management endeavors shows promising results in the biocontrol of *Pythium* spp., as well as their positive impact on promoting the growth of solanaceous crops (Kenawy et al., 2019), and many other major crop varieties. In many cases, it has been properly noted that imported bioformulations occasionally exhibit suboptimal performance owing to the influence of climate change (Compant et al., 2010) and limitations in nutrient availability (Kandeler et al., 2006). Therefore, the detection and characterization of PGPR endemic to chili rhizospheres capable of suppressing the inoculum of *P. myriotylum* and enhancing the growth of chilies in nurseries is in need.

### 7.4 *Trichoderma* and *Bacillus* as a biocontrol agent against *Pythium* spp

*Trichoderma*, a prominent genus of filamentous fungi within the phylum Ascomycota, demonstrates remarkable potential as a biocontrol agent (BCA) against *Pythium* pathogens (Thambugala et al., 2020; Yao et al., 2023). These beneficial fungi, particularly the avirulent strains, have proven effective in plant protection, biostimulation, and biofertilization. Their effectiveness in agricultural applications depends on their metabolic activity and interactions with plants and other microorganisms.

*Trichoderma* species can colonize the rhizoplane and plant roots (Poveda and Eugui, 2022). They produce an array of metabolites with antimicrobial properties, including cell wall-degrading enzymes, both volatile and non-volatile antibiotics (Jayaraj et al., 2006), as well as phytohormones and phytoregulators such as Indole Acetic Acid (IAA), cytokinin and ethylene that stimulate plant growth indirectly (Rahman et al., 2012). They also secrete chitinase, which enhances plant defense mechanisms (Subash et al., 2013) against fungal pathogens, and 1–3 glucanase, a protease contributing to fungal pathogen defense and improved nutrient supply (Mannai and Boughalleb-M’Hamdi, 2023). Conversely, *Trichoderma* stimulates plant resistance locally or systemically by releasing products known as elicitors (Majeed et al., 2019). These elicitors originate from the cell walls of both the host plant (endoelicitors) and the invading microorganism (exoelicitors). *Pythium* acidifies soil by secreting organic acids that can solubilize phosphate and other micronutrients supporting plant nutrition (Rahman et al., 2012).

Notably, *Trichoderma* can control *Pythium* spp. through mechanisms such as necrotrophic mycoparasitism, competition for nutrients and space, and the synthesis of antifungal metabolites (Tyśkiewicz et al., 2022). These fungi exhibit rapid growth, metabolic versatility, and resistance to various toxic chemicals, including fungicides and herbicides, as adaptations (El Enshasy et al., 2020; Jia et al., 2024; Yang et al., 2024).

Among *Trichoderma* species, *T. harzianum* strains stand out for their ability to protect plants by antagonizing *Pythium* pathogens in damping-off (Muthukumar et al., 2011b), showing more promising performance than *T. viridae, B. subtilis,* and *T. asperellum,* which exhibit synergistic effects against *P. aphanidermatum* in solanaceous crops (Kipngeno et al., 2015). Peroxidase activity was significantly higher in the roots of sugar beet seedlings treated with *T. virens* as biocontrol agents (Hanson and Howell, 2004).

*Pseudomonas fluorescens* is a beneficial bacterium widely used as a biocontrol agent against damping-off disease in chili via antibiosis, siderophore production, competition, induced systemic resistance, and enzyme production against damping-off disease in chili (Mehmood et al., 2023). Bacterial species such as *Bacillus* have been proven to control fungal diseases. *B. subtilis* showed high antagonistic activity against *Colletotrichum gloeosporioides*, which caused anthracnose disease of chili (Ashwini and Srividya, 2014). *Bacillus* has chitinolytic activity to control different fungal pathogens. Chitinolytic is a pathogenesis-related protein that increases the plant’s defense mechanism against fungal pathogens (Mabuchi et al., 2000; Huang et al., 2005). *Bacillus* species were found to colonize the root surface, increase plant growth, and cause the lysis of fungal mycelia (El-Bendary et al., 2016). As shown in previous reports, *Bacillus* spp. significantly control the damping-off chili caused by *P. myriotylum* (Jimtha et al., 2016). Similar results from Hyder et al. (2021) showed that *B. cereus* and *B. megaterium* significantly control damping off disease (Hyder et al., 2021).

### 7.5 Use of resistant varieties

Recent advances in plant breeding have significantly improved efforts to develop resistant chili cultivars to combat biotic and abiotic stresses. Marker-assisted selection (MAS) has accelerated the identification and incorporation of resistance genes into new cultivars, allowing breeders to develop varieties resistant to diseases such as powdery mildew, anthracnose, bacterial wilt, and viral diseases. Quantitative trait loci (QTL) mapping has been crucial in identifying resistance traits linked to these pathogens. Additionally, genomic selection and CRISPR-Cas9 gene editing technologies are revolutionizing breeding by enabling the precise modification of specific genes responsible for resistance. Traditional methods like germplasm screening and hybridization remain integral, but these are now supplemented with advanced genomic tools to improve efficiency and precision.

Host plants have evolved complex defense mechanisms that can either activate or suppress a range of genes when under attack by pathogens (Kumar et al., 2022). These induced resistance responses include various physiological processes such as the generation of reactive oxygen species, synthesis of phytohormones, and production of pathogenesis-related (PR) proteins. The utilization of host plant resistance as a management strategy encompasses two distinct approaches: (i) the utilization of plant varieties that possess resistance to pathogens, resulting in reduced pathogen populations or enhanced ability to resist pathogen-induced damage, and (ii) the integration of these resistant varieties with other management tactics as part of an integrated pest management (IPM) framework. However, a significant number of plant diseases, especially those responsible for damping-off diseases, lack any plant cultivar that exhibits quantifiable resistance (Babadoost and Islam, 2003). As a result, the optimal utilization of crop varieties possessing pathogen resistance can only be achieved by effectively integrating them with other strategies for disease management. However, there has been a lack of emphasis on incorporating plant resistance into other IPM strategies and a lack of research on quantifying the benefits of plant resistance within IPM programs that utilize various approaches (Davis, 2009).

The current breeding methodology employed thus far for the development of crop varieties that exhibit resistance should be reevaluated if the intent is to prioritize sustainable crop protection strategies based on IPM. This is especially valid because the majority of cultivated plant varieties developed thus far have been rooted in a market-oriented strategy emphasizing the cultivation of high-yielding and economically advantageous crop varieties. The current tendency has resulted in increased utilization of short rotations or monoculture methods while neglecting the potential benefits that minor crops may offer for IPM (Messéan et al., 2016). Crop diversification is hindered by the limited range of minor crop types, which limits beneficial activities like multiple cropping and intercropping (Benvenuto et al., 2020). Hence, in light of the pivotal role of breeding in enhancing crop competitiveness and facilitating adaptation to diverse cropping systems, it is imperative to use a distinct approach for breeding in IPM compared to conventional methods (Benvenuto et al., 2020). Detailed information about management strategies is described in Table 4.

**Table 4:** Control measures for managing damping-off in chili reported from different parts of the world.

Species targeted	Active ingredients	References
Chemical control		
*Pythium aphanidermatum*	Propamocarb	Male and Vawdrey (2010)
*P. ultimumin*	Carbendazim and copper oxychloride	Singh et al. (2019)
*P. ultimumin*	metalaxyl/mefenoxam	Buchenau et al. (1981)
*Pythium* spp.	Metalaxyl, cyamoxanil, and carbendazim	Zagade et al. (2016)
*P. ultimum*	Metalaxyl	Zagade et al. (2014)
*P. ultimum*	Benlate	Ansari et al. (1990)
*P. ultimum*	Metalaxyl and Captan	Gholve et al. (2014)
*P. ultimum*	Carbendazim and Mancozeb	Gholve et al. (2014)
*P. aphanidermatum*	Thiram 75 WS and Captan 50 WP	Saha et al. (2011)
*P. aphanidermatum*	Metalaxyl	Zagade et al. (2016)
*P. ultimum*	Metalaxyl	Zagade et al. (2016)
*P. ultimum*	Ethaboxam	White et al. (2019)
*P. ultimum*	Ethaboxam	Wang et al. (2021)
*P. graminicola*	Carboxin and Thiram	Dubey et al. (2019)
Biological control		
*P. aphanidermatum*	*T. viride* and *P. fluorescens*	Muthukumar et al. (2011a)
*P. aphanidermatum*	*T. viride* and *P. fluorescens*	Muthukumar et al. (2010)
*P. aphanidermatum*	*Calothrix elenkenii*	Manjunath et al. (2010)
*P. ultimum*	*Stenotrophomonas rhizophila*	Lara-Capistran et al. (2020)
*P. ultimum*	*B. subtilis*	Lara-Capistran et al. (2020)
*P. debaryanum*	*B. subtilis*	Ali et al. (2016)
*P. aphanidermatum*	*T. harzianum*	Mehetre and Kale (2011)
*P. ultimum*	*Cryptococcus laurentii*	Muthukumar et al. (2011b)
*P. aphanidermatum*	*Streptomyces griseoviridis*	Punja and Yip (2003)
*P. ultimum*	*Gliocladium catenulatum*	Mcquilken et al. (2001)
*P. myriotylum*	*P. putida*	Hyder et al. (2021)
*P. aphanidermatum*	*Exiguobacterium indicum*	Al-Hussini et al. (2019)
*P. aphanidermatum*	*Actinoplanes campanulatus*	El-Tarabily et al. (2009)
Physical control		
*Pythium* spp.	Tillage	Bailey and Lazarovits (2003)
*Pythium* spp.	Cover crops and soil residue management	Bailey and Lazarovits (2003)
*Pythium* spp.	Crop rotation and intercropping	Hwang et al. (2008)
*Pythium* spp.	Moderate humidity and avoid waterlogging, adequate light, and optimal temperatures	Schmidt et al. (2004)
*Pythium* spp.	Phosphorus, potassium, and calcium	Landis (2013)
*Pythium* spp.	Avoid excessive plant densities	Landis (2013)
*Pythium* spp.	Proper sowing date and good drainage	Hwang et al. (2000)
*Pythium* spp.	Soil pH	Lamichhane et al. (2017)
*Pythium* spp.	Seedbed preparation	Njoroge et al. (2008)
*Pythium* spp.	Seed quality	Roberts et al. (2016)

### 7.6 Detrimental effects of synthetic fungicides

Even though chemical fungicides are effective in rapidly controlling damping-off, they lead to human and ecotoxicity, particularly in developing nations (Voorrips et al., 2004). Human toxicity includes irritant dermal injuries, mucus membranes, and dermal sensitization. For animals, livestock poisoning and aquatic organisms, particularly fish, experience adverse impacts from fungicides. Furthermore, fungicides reduce the fungal and total microbial biomass in soil (Verdenelli et al., 2023), influencing the imbalance in soil microbiota, and another detrimental effect is phytotoxicity by fungicide residues (Kovačič et al., 2013). Different fungicides have different modes of action and differ in how long they last to suppress disease. At the same time, fungicide resistance occurs with the longer usage of the same fungicide type. For a greater chance of enhanced disease protection in the fields, it is strongly advised to rotate two or more distinct classes of fungicides to prevent the development of fungicide-resistant pathogens (Förster et al., 2007). It is hard to achieve sustainable disease management by using fungicides alone. Hence, the need for integrated disease management is discussed.

### 7.7 Advances in molecular aspects of detection as well as management of damping-off diseases

Recent advances in the molecular understanding of damping-off diseases have significantly enhanced detection and management strategies. Molecular diagnostics, such as polymerase chain reaction (PCR) and quantitative PCR (qPCR), now allow for rapid and precise identification of soil-borne pathogens like *Pythium*, *Fusarium*, and *Rhizoctonia* at early stages of infection. These techniques enable species-level identification and quantification of pathogen load in soil and plant tissues, which can help predict disease outbreaks. The advent of metagenomics and next-generation sequencing (NGS) has further revolutionized detection by allowing a comprehensive analysis of microbial communities, identifying emerging pathogens, and understanding the microbial shifts that occur during disease progression (Fu et al., 2024).

On the management side, molecular advances have improved the use of biological control agents and the development of resistant cultivars. Functional genomics, through tools like RNA interference (RNAi) and CRISPR-Cas9, is used to knock out or modify plant susceptibility genes, providing a novel method for increasing disease resistance. Furthermore, insights into the molecular interactions between pathogens and biocontrol agents, such as *Trichoderma* and *Pseudomonas fluorescens*, have led to enhanced formulations that can better suppress pathogen activity through targeted modes of action, such as mycoparasitism and antibiosis. These advances highlight the critical role of molecular tools in both early detection and integrated management approaches, offering a promising future for sustainable control of damping-off diseases.

## 8 Conclusion

Damping-off disease remains a formidable challenge in chili cultivation, further exacerbated by environmental changes and the advent of more aggressive pathogen strains. Our comprehensive examination reveals that integrated disease management is the key to addressing this issue. While chemical fungicides provide immediate control, their long-term usage is unsustainable due to environmental and human health concerns as well as the potential for resistant pathogen strains to develop. Sustainable management of damping-off requires a multifaceted approach, including the development of resistant chili varieties, the implementation of strategic cultural practices, and the adoption of novel technologies such as nanoparticles. These strategies must be adaptable to evolving climatic conditions and tailored to local agro-ecological contexts.

Moreover, there is a pressing need for further molecular studies to enhance our understanding of plant–microbe interactions, which could lead to more effective and targeted control measures. The innovative application of nanoparticles in the laboratory setting shows great promise and could pave the way for new biocontrol methods that are both effective and environmentally sustainable. Ultimately, a comprehensive integrated pest management framework that combines traditional practices with cutting-edge research is essential for the holistic and sustainable management of damping-off disease in chili cultivation. As we advance our scientific knowledge and integrate it into field-based applications, we move closer to safeguarding the future of chili production against the persistent challenges posed by this destructive disease.

### 8.1 Future prospects

The challenge of damping off disease remains as critical as ever, especially with its large-scale spread exacerbated by flooding and climate change. Despite extensive research on the epidemic nature of the disease, gaps still persist in our understanding of host–pathogen interactions, disease proliferation, and effective management strategies.

There is an urgent need to devise efficient integrated management strategies that consider the increasing incidence of flooding, environmental factors, and the variability of pathogenic races. Developing resistant varieties of chili appears to be one of the most promising approaches for the long-term management of damping-off disease. By breeding for resistance, we can reduce the vulnerability of crops to this disease under various environmental conditions.

In addition to developing resistant varieties, integrating cultural practices tailored to specific climatic changes is crucial for more effective disease management. As the climate continues to change, previously effective strategies may need to be adapted to remain effective under new conditions. Furthermore, there is a growing need for more molecular studies to deepen our understanding of plant–microbe interactions. Insights into the infection mechanisms of pathogens will be invaluable for creating targeted management strategies that can interrupt the infection process and mitigate the disease’s impact.

Additionally, our laboratory is exploring the potential of nanoparticles as a novel approach to combat *Pythium* spp. Nanoparticles represent an innovative frontier in managing damping-off disease in chili. These microscopic particles could offer new mechanisms of action against the pathogen, potentially enhancing the effectiveness of existing treatments or providing alternative management solutions. As this research advances, it could open new pathways for controlling this persistent and damaging disease.

## Supplementary material

Supplementary Figure S1. The botanicals characteristics of chilli plant. **(a)** Chilli plant. **(b)** Ripe red fruit. **(c)** Immature green fruits. **(d)** Ripe red fruit.

Supplementary Figure S2. Characteristics symptoms of damping-off disease on young seedlings of chilli plants. **(a)** Symptoms on young seedlings. **(b)** Whitish growth on the stem near soil surface. **(c)** Diseased plants wilted after severe attack. **(d)** Healthy plants.
