# Supplementary figures and images for: A comprehensive review of integrated management strategies for damping-off disease in chili
Source: Front Microbiol. 2024 Oct 17;15:1479957. doi: 10.3389/fmicb.2024.1479957 (PMC11524829; doi:10.3389/fmicb.2024.1479957)

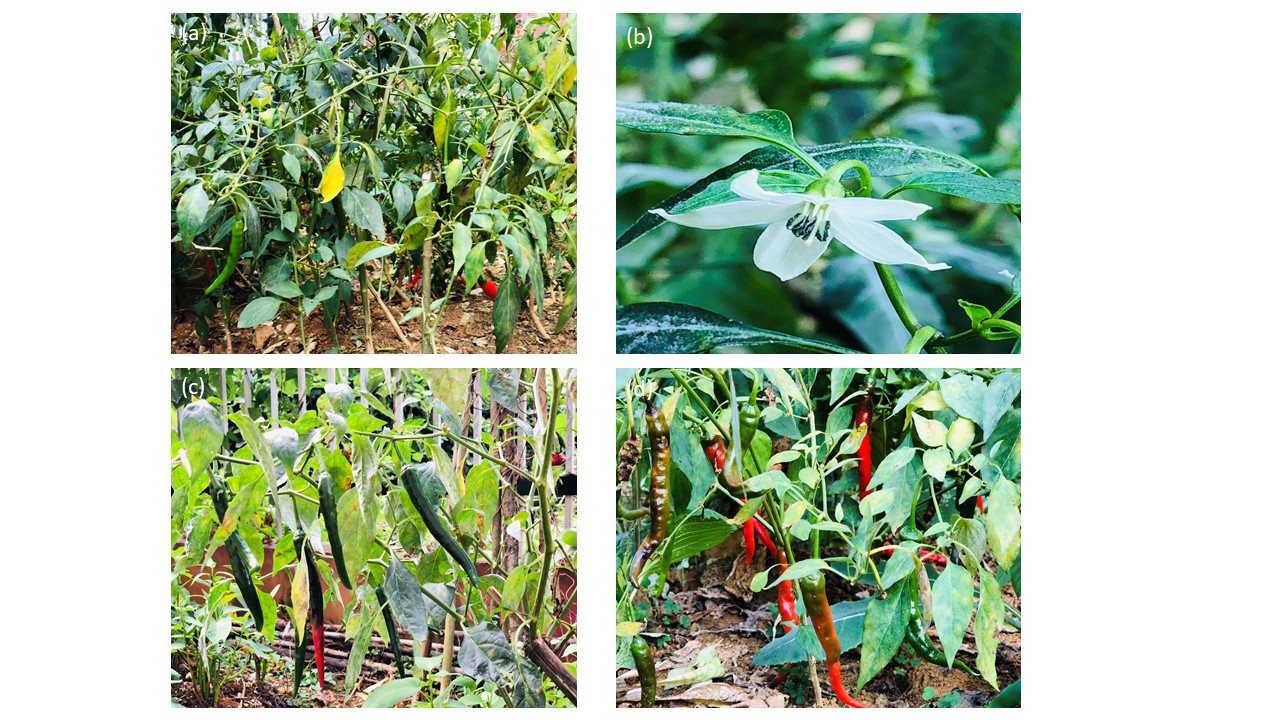

Supplement: Supplementary Figure S1. — The botanicals characteristics of chilli plant. (a) Chilli plant. (b) Ripe red fruit. (c) Immature green fruits. (d) Ripe red fruit. [file Image_1.JPEG]

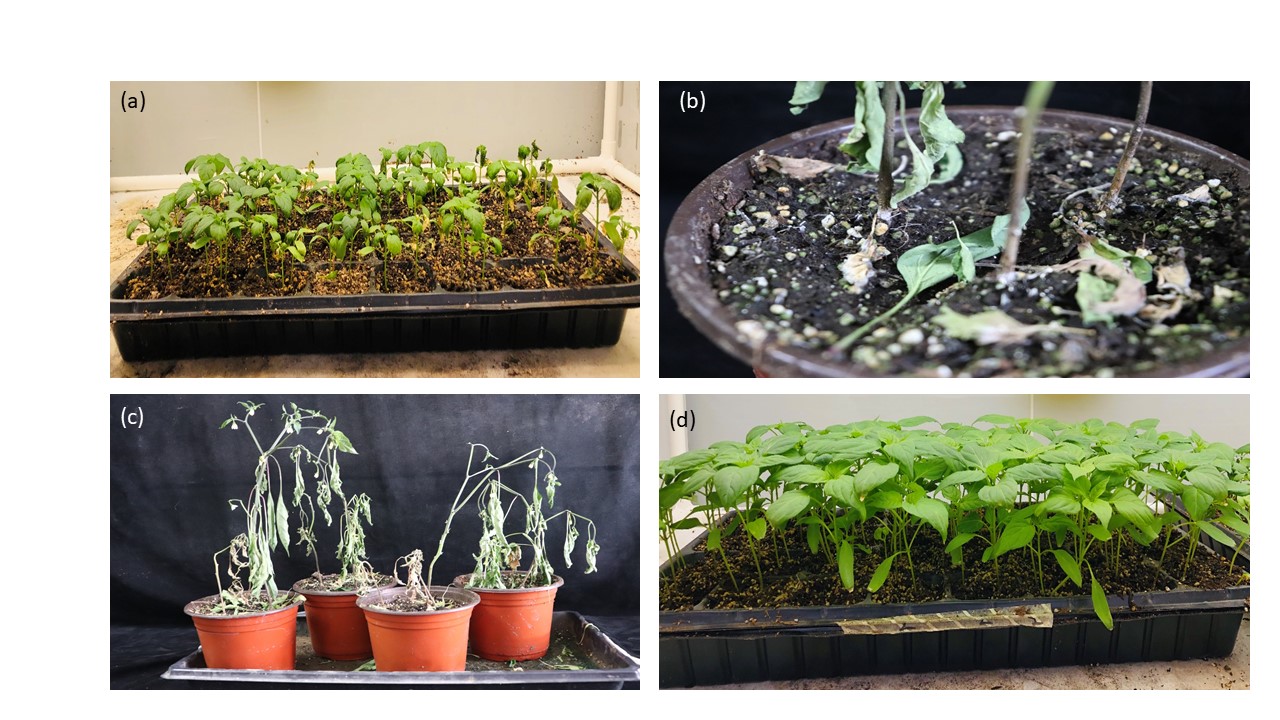

Supplement: Supplementary Figure S2. — Characteristics symptoms of damping-off disease on young seedlings of chilli plants. (a) Symptoms on young seedlings. (b) Whitish growth on the stem near soil surface. (c) Diseased plants wilted after severe attack. (d) Healthy plants. [file Image_2.JPEG]
